# Deaths from Inhalation of Sulphuric Ether

**Published:** 1849-07

**Authors:** 


*Deaths from Inhalation of Sulphuric Ether.* By Paul F. Eve, M. D., Prof. of Surgery in the Med. College of Georgia.

Case I. Mr. J., a member of the class in our College the past winter, and a candidate for the degree in medicine, inhaled sulphuric ether during the evening of the 3d of last March. The article was obtained from a druggist of good reputation, in quantity two ounces, and the motive for using it, was its exhilarating effects, which he had experienced before. It was inhaled from a pocket-handkerchief, renewing or applying it three times, and about one ounce was supposed to have been consumed. The time of inhaling it was reported to be considerable, and a companion of Mr. J. removed the handkerchief suddenly while he was still breathing it. He became then furiously excited, and it required several persons to control him. He was forced upon a bed, where he soon fell asleep. A few moments afterwards, another student of medicine, not liking his breathing, which he reported to be sonorous, awakened him, when he again became much excited; indeed, so much so, that cold water was dashed over him. He now retired to bed, and nothing special was noticed until the next morning. He awoke perfectly rational, but complained of great pain in the forehead. This continuing unabated, I was sent for to see him at 2, P. M., on the 4th. Magnesia and salts in purgative doses, cold applications to the head, mustard-plaster to the neck and warm pediluvia were prescribed; with the expression of the hope that these means would give entire relief I was again sent for at 8, P. M., and also at 8, A. M.. of the 5th, (the next day,) but did not see the patient until 11 o'clock, three hours after; he had been visited, and prescribed for in the meantime by Drs. Carter and Dugas; Dr. Ford was subsequently added to the consultation. Symptoms of meningitis, &c., persisted in spite of all treatment pursued, and our patient died on the morning of the 7th.

Case II. For this I am indebted to a friend:—During a recent visit to Huntsville, Alabama, among the several excellent professional brethren I met with there, was Dr. John Y. Bassett, who, among other advantages, had visited Europe. At my request, he kindly furnished the particulars of a case of *tetanus* to which he was called on the 15th of August, 1847. In the progress of it, Dr. Fearn, whose reputation is well known throughout our country, and who has twice been elected to a professorship in our Medical Colleges, was called into consultation. He proposed the actual cautery and the inhalation of sulphuric ether. Dr. B. says, at this time the patient's "pulse was good and there were no signs of immediate extinction of life. I heated my cautery, and sent for a Dentist who was in the habit of administering the ether. I gave a watch to the owner of the negro affected with lock-jaw, and requested him to speak at every quarter of a minute. In one minute, the patient was under its influence; in a quarter more he was *dead*—beyond all my efforts to produce artificial respiration or restore life." All present thought he died from inhaling the ether.

Of course these cases should by no means be used as objections to the judicious employment of etherization. They are only adduced as proofs to the position, that *ether* as well as chloroform may produce death.—*Southern Med. and Surg. Journal.*

